# Approach Direction Prior to Landing Explains Patterns of Colour Learning in Bees

**DOI:** 10.3389/fphys.2021.697886

**Published:** 2021-12-08

**Authors:** Keri V. Langridge, Claudia Wilke, Olena Riabinina, Misha Vorobyev, Natalie Hempel de Ibarra

**Affiliations:** ^1^Department of Psychology, Centre for Research in Animal Behaviour, University of Exeter, Exeter, United Kingdom; ^2^Department of Psychology, University of York, York, United Kingdom; ^3^Department of Biosciences, Durham University, Durham, United Kingdom; ^4^School of Optometry and Vision Science, University of Auckland, Auckland, New Zealand

**Keywords:** insects, colour vision, pattern vision, learning, insect flight, behaviour, flower patterns

## Abstract

Gaze direction is closely coupled with body movement in insects and other animals. If movement patterns interfere with the acquisition of visual information, insects can actively adjust them to seek relevant cues. Alternatively, where multiple visual cues are available, an insect’s movements may influence how it perceives a scene. We show that the way a foraging bumblebee approaches a floral pattern could determine what it learns about the pattern. When trained to vertical bicoloured patterns, bumblebees consistently approached from below centre in order to land in the centre of the target where the reward was located. In subsequent tests, the bees preferred the colour of the lower half of the pattern that they predominantly faced during the approach and landing sequence. A predicted change of learning outcomes occurred when the contrast line was moved up or down off-centre: learned preferences again reflected relative frontal exposure to each colour during the approach, independent of the overall ratio of colours. This mechanism may underpin learning strategies in both simple and complex visual discriminations, highlighting that morphology and action patterns determines how animals solve sensory learning tasks. The deterministic effect of movement on visual learning may have substantially influenced the evolution of floral signals, particularly where plants depend on fine-scaled movements of pollinators on flowers.

## Introduction

Eyes and associated neural architectures have evolved in diverse ways to provide animals with adaptive views of the world. Viewing conditions are also shaped by gross morphology, since the structure of the head and body will dictate the extent of coupling between vision and movement (Land, 1999). Where the eyes and/or head are highly mobile, as in many terrestrial vertebrates, viewing direction and thus acquisition of visual information can be uncoupled from body movement—the animal can look all around while, for example, moving forward. In contrast, other animals cannot shift their gaze independently from the body due to morphological constraints, meaning that the direction of gaze is more closely tied with their actions. This coupling is particularly pronounced in many insects which are capable of very limited head movements and lack the ability to move their eyes and lenses within the head. Although flying insects, like bees and flies, display fast and minute head movements during flight to stabilise gaze (e.g., Schilstra and van Hateren, 1998; Boeddeker et al., 2010; Riabinina et al., 2014), to vary their viewing direction and field of view to detect image features they must change the orientation and position of the entire body. Thus, the gross movement of the insect dominates its experience of the visual environment. Insects can actively alter movement patterns to change viewpoints and actively acquire visual information (e.g., Lehrer and Srinivasan, 1994; Land and Collett, 1997; Egelhaaf et al., 2012), but this has consequences for the behavioural task at hand, and could therefore incur costs to an extent that will determine when and how active vision strategies are employed. Here we address a little explored scenario where the efficient execution of action sequences takes a high priority in a behavioural task, asking whether that will significantly influence an insect’s perception of readily visible objects.

Motor performance can be costly, even though it may appear to the human observer that an animal moves effortlessly. For instance, air is a very viscous medium for flying insects and they have to obey the laws of aerodynamics, hence insect flight, and landing manoeuvres are complicated and require a number of well-coordinated actions (Dickinson et al., 2000; Fry et al., 2005; Vance et al., 2014). Bees landing on a horizontal or vertical surface exhibit a sequence of highly stereotyped visually controlled movements in order to alight successfully (Srinivasan et al., 2000; Baird et al., 2013; Reber et al., 2016). We argue that, when visual cues are not limiting, efficient motor patterns define the viewing conditions and incidentally determine what visual information is acquired for solving various behavioural tasks, such as learning the colours and patterns of a food source, a task of particular importance for bees foraging on flowers.

Colour and pattern perception in bees has been widely investigated, and it has been often assumed, implicitly and sometimes explicitly, that bees will adopt movement patterns that optimally support solving the perceptual learning tasks, e.g., approach a target in the most convenient way to view all available visual features (e.g., Wehner, 1972; Menzel and Lieke, 1983; Lehrer, 1998; Giurfa et al., 1999a; Hempel de Ibarra et al., 2002; Thivierge et al., 2002; Zhang et al., 2004; Dyer et al., 2005; Wu et al., 2013; Avarguès-Weber et al., 2020). However, the viewing conditions of individual bees will be wholly dependent upon their flight behaviour during approach and landing on the stimulus (Hertz, 1935; Wehner and Flatt, 1977; Giurfa et al., 1999b). Previous evidence suggests that bees might generally prefer to approach vertically presented stimuli from below (Anderson, 1977; Giger, 1996), which could significantly affect perception and learning processes. This question is highly relevant for understanding the bee’s natural foraging behaviour, as many flowers are tilted or vertically oriented. We show that the outcome of a learning task with flower-like colour patterns is indeed determined by the bees’ approach directions during training.

## Materials and Methods

### Setup

Experiments took place indoors between October 2011 and March 2013, using bumblebees (*Bombus terrestris* Linnaeus 1758) from six colonies supplied by Koppert United Kingdom Ltd. Bees were housed in the nest box provided by the breeder. They could access a Plexiglas exit box and from there two tunnels through doors that could be opened and closed by the experimenter in order to separate bees. Only one marked bee was allowed to enter the tunnel (the “flight tunnel”) that led to the experimental flight cage at any one time (mesh netting on a Dexion frame: 110H×80L×80W cm). The other bees were either waiting in the exit box or deviated into the second tunnel that led to a small box. On the far end of the cage, opposite to the tunnel exit, a vertical plastic wall (20 × 20 cm) displayed the coloured target on a grey background (Supplementary Figure 1). The centre of the target was aligned in height with the centre of the exit tunnel. At the end of a trial, the bee was gently caught and placed back into the exit box, so that it could return to the nest. The flight cage was predominantly lit by natural daylight from a large window wall, but in addition the lab’s high-frequency lighting and three 36W diffused strip lights above the flight cage were switched on. A video camera (Photron SA-3 or Panasonic SDR-H90) was positioned perpendicular to the vertical stand with the target to record a bee’s approach from the side over the last 10 cm during training and test trials. In some of the test trials (when the test pattern’s contrast line was rotated by 90°) the bee’s choice behaviour was recorded by a second video camera from above.

### Stimuli

Target stimuli, colour discs or bicolour patterns (8 cm diameter), and their grey background were printed on a single sheet, centred and glued to the front of a 20 × 20 cm plastic stand. A transparent pipette nib (4 mm diameter) inserted in the centre of the stimulus was backfilled with 50% sucrose solution. The nib was a short protrusion and inconspicuous, as we know from our previous studies investigating the spatial resolution of bee vision with different colours and coloured patterns, and showing that bees easily learn the colours of large patterns from a distance (e.g., Giurfa et al., 1996; Giurfa et al., 1999b; Hempel de Ibarra et al., 2001; Wertlen et al., 2008). The nib was filled with sucrose solution during training trials, and empty during test trials. Eight different training patterns were used: a plain disc (Yellow or Blue) or bicolour patterns, either with the horizontal contrast line in the centre or with the contrast line below or above the centre. Each bee was trained with only one of these stimuli.

When the contrast line was in the centre of the bicolour pattern, both colour parts were equal in size. Patterns are henceforth named with the colour acronym for the colour at the top followed by the one for the colour at the bottom. When the yellow colour was presented in the top half, the bottom half of the same size was blue (pattern Y:B), and vice-versa, when the blue colour was in the top, the yellow colour was in the bottom (pattern B:Y). The rewarding nib was located in the middle of the contrast line during training trials.

When the position of the line was shifted to change the size of the colour parts in a different set of training patterns, the line was either at a quarter of the disc’s diameter above the bottom or below the top of the disc (3:1 diameter ratio). In these 3:1 patterns the reward was also offered in the centre of the whole pattern. However, since the colour parts were unequal in size, this meant that it was always located inside the large colour part. When the contrast line was near the bottom of the disc, the upper large part of the pattern was blue and a small section displayed at the bottom (3B:1Y), whilst the reward was delivered in the centre of the whole pattern. The second pattern displayed a larger upper part in yellow with the small bottom section in blue (3Y:1B). However, when the contrast line was positioned in the upper half of the disc, the smaller section was at the top. Again, the reward was delivered in the centre of the pattern. The resulting pattern displayed an upper small segment in yellow above a large blue segment (1Y:3B), or, correspondingly, a small blue segment above the large yellow segment (1B:3Y). Blue and yellow were chosen because they are easily learnt and discriminated by bees, as confirmed by our spectral measurements (see Hempel de Ibarra et al., 2014 for details). The colours differed from each other and from the grey background in terms of both brightness and chromatic contrast for the bee eye under the illumination conditions of the flight cage.

### Training Procedures

Two experiments were completed. In Experiment 1, a bee was trained to either a single-coloured disc (Yellow, *N* = 10 bees, Blue, *N* = 10 bees) or a bicolour pattern with equal-sized parts (B:Y, *N* = 11 bees, Y:B, *N* = 12 bees). In Experiment 2, other bees were trained, each with only one of four bi-colour patterns in which the contrast line was shifted (3B:1Y, 3Y:1B, 1B:3Y, or 1Y:3B, *N* = 10 bees in each group).

We recorded and analysed the bees’ approaches over a distance of 10 cm. The visual angle subtended by the target varied from 40° to above 150° as the bees approached for landing. The bees’ speed decreased during the approach to the target. The drop was very apparent when the bees were reached the distance of 4 cm and less from the target [average speeds ranged 16.3–24.0 cm/s (>4 cm) and 4.9–8.4 cm/s (<4 cm)]. There were no significant differences in the average approach speed between groups (Supplementary Figure 3).

Whilst the bees tend to pitch the abdomen much more frequently, the head’s pitch appears to be kept very steady relative to the horizontal flight direction toward the target (K. Langridge and N. Hempel de Ibarra, personal observations from high-speed video footage). Only in the very last moments of the landing manoeuvres when the bees tilt their body to position the legs at the target, the head is pitched upwards in synchrony with the rest of the body. The bees would typically extend the proboscis while they landed, therefore we did not analyse any landing or drinking responses. In the final part of the approach, just prior to landing, the bees’ antennae came close or even in contact with the nib, and we must assume that they possibly see it and sense the humidity from sucrose solution in the small opening (Harrap et al., 2021). We therefore excluded any data points closer than 0.5 cm from the analysis of the trajectory data.

All bees were marked and pre-trained individually over a few trials, typically 1–3 trials, to fly to the target stand (20 cm × 20 cm) that was covered by a uniform grey background and positioned 60 cm away from the flight tunnel entrance, before switching to a new one displaying the coloured pattern and the grey background (Supplementary Figure 1). Individuals were given two further practice flights before filming began.

**FIGURE 1:**
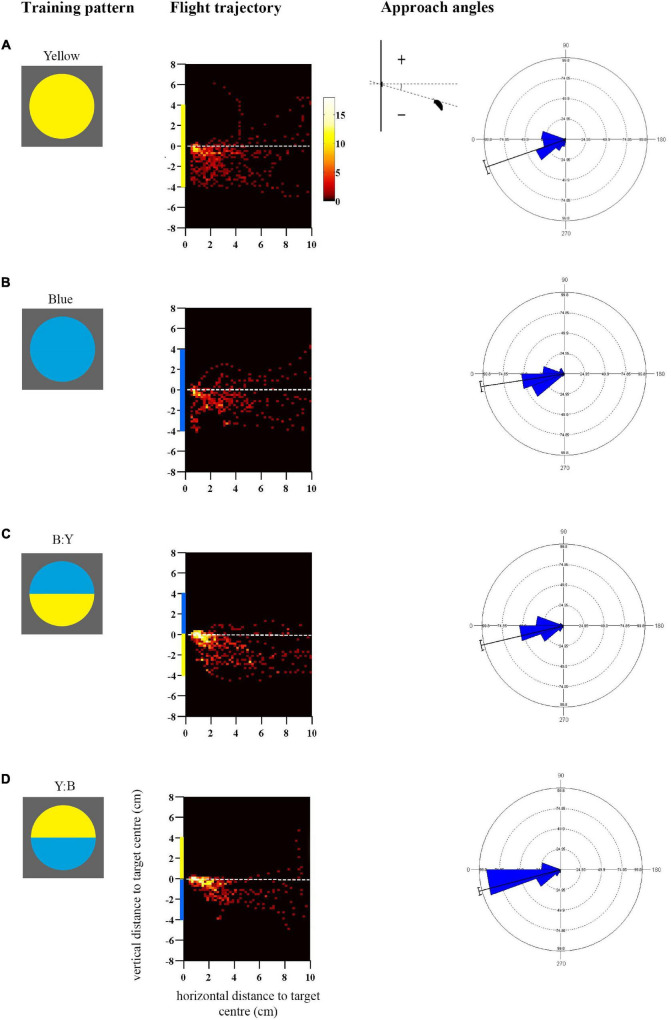
Approach flights of bumblebees trained to collect sucrose from the centre of a vertically presented coloured disc, over a distance to the target from 10 to 0.5 cm. Training patterns were single-colour **(A)** Yellow or **(B)** Blue discs, or bicolour **(C)** B:Y or **(D)** Y:B patterns with a central contrast line, presented on a grey background. Heat-map plots depict the video capture area (filmed from the side) divided into 0.04 cm2 pixels denoting the frequency of positions of bees during the last training flight prior to tests (see colour scale next to **A**) (*N* = 10 bees for **(A,B)**; **(C)** *N* = 11 bees and **(D)** *N* = 12 bees). *X*-axis depicts horizontal distance and *Y*-axis vertical distance to the target plane. Approach angles are shown on the right (see inset). Angles between 0° and 270° indicate a position below the contrast-line, and angles between 0° and 90° above the contrast line.

Each bee was only trained with one type of training stimulus of eight coloured targets described above. During 10 rewarded consecutive trials each bee was filmed flying toward, and landing on, the training stimulus. Sometimes, bees explored the upper half of the flight cage during this training, rather than immediately initiating the approach flight from the tunnel exit. They would, however, descend voluntarily at a distance from the target, typically in the middle of the cage or the area closer to the tunnel and initiate the approach flight toward the target (see also Figure 1 and Supplementary Figure 2). Only one bee was ever present in the flight cage at any one time. Bees from several colonies participated in all colour treatments. Nibs and paper stimuli were replaced frequently (every few trials) to preclude any potential build-up of olfactory cues.

### Colour Learning Tests

On completing training, each individual was subject to unrewarded tests in order to examine how the colour targets were learned. As during training, there was only one bee present in the flight cage during each trial. The duration of each test trial was 3 min. An empty nib was located in the middle of the central contrast line. Since bees could not sense the presence of the reward, they did not attempt to land on the nib but would search flying in front of the stimulus. This slower searching flight behaviour was observed within a distance of 5 cm. Test trials were separated by 1–3 refreshment trials with the rewarded training target to maintain the foraging motivation of the bees. The test sequence was varied across individuals. Since the test conditions were identical for the different groups and the bees did not attempt to land on the empty nib, any variation seen in test responses indicates differences in the formation of colour preferences during the approach flight in the training trials.

In a test trial the bee was presented with one of the test patterns: a single disc partitioned by either a horizontal or a vertical central boundary into two equally sized segments, one blue and one yellow. Bees trained to single-colour stimuli had two tests with such bicolour patterns where the coloured segments were separated by a horizontal contrast line (B:Y and Y:B). Bees trained to patterns with a horizontal central contrast line were given three separate tests with bicolour test patterns that resembled the training pattern but were rotated by 90°, 180°, and 270°. Bees trained to patterns with an off-centre contrast line had two tests with bicolour test patterns with a horizontal contrast line (B:Y, Y:B) and one test with a vertical contrast line (Blue on the right, Yellow on the left).

Test duration was 3 min from the moment of release into the flight cage. Tests with patterns where the central contrast line was horizontally oriented were filmed from the side, while test patterns with a vertical contrast line were filmed from above, such that the bees’ choices between the two colour segments could be compared. All bees completed all training and test trials, with the exception of two individuals, which completed two tests each.

### Data Analysis

For each bee, its approach during training was clipped from the video footage (25 fps) over the distance of 10–0.5 cm from the target. The approach height of the bee relative to the dorso-ventral arrangement of the colour patterns was extracted from clipped footage using a Matlab routine (see Hempel de Ibarra et al., 2009 for details), recording the position of the top line of the bee head (guided by the bee’s antennae base) in relation to the centre of the target (Figure 1).

Approach angles are defined as the angle between the head position of the bee and the contrast line within bicolour patterns, or the central horizontal line of the target for single-coloured targets, relative to the horizontal direction of approach (see inset in Figure 1A). We standardised the data by extracting the first approach angle when the bee entered a new distance bin of 0.5 cm during the approach. The approach angles were analysed using Mardia-Watson-Wheeler multiple comparisons tests in a circular statistics programme (Oriana V.3). Approach speeds were determined and averaged over the distance of 10 cm until the bee crossed 0.5 cm.

Three-minute learning tests were analysed using the behavioural data-logging freeware JWatcher^1^ (Blumstein and Daniel, 2007), which calculated the total search time of the bees on the blue or yellow sector when flying in front of a stimulus (over a distance of 5 cm). Search time was defined as the time the bee spent flying in front of the test stimulus and visually exploring it, within 5 cm distance, facing toward the pattern, during a 3-min test. Searching of the top vs. the bottom colour-half was determined by the position of the top of the bees’ head relative to the contrast line.

Statistical analysis of linear data was carried out using SPSS and Matlab. Data met the assumptions of parametric tests unless otherwise stated. Paired *t*-tests and Wilcoxon tests, if the assumption of normality was not met, were used to compare the search times of bees on the segments of the bicolour patterns. The amount of time a bee spent flying below the centre of a bicolour patterns over ten training flights, and the strength of their preference for the colour of the lower half was tested using Spearman Ranks correlations.

## Results

### Experiment 1: Single-Colour and Bicolour Patterns With a Central Contrast Line

#### Approach Flights During Training

Bees trained to collect sucrose from the centre of a vertically presented coloured disc preferentially directed their approach toward the lower half of the pattern prior to landing (Figure 1; see also Supplementary Figures 2A,B for the mean heights of the flight trajectories in the last training flight). The flight trajectory heat maps in Figure 1 illustrate the remarkable consistency of this behaviour in bees that were not experimentally restrained in their direction of approach and landing, in response to both single-coloured discs (Figures 1A,B) and bicolour patterns (Figures 1C,D). The trajectories suggest that the approach from below-centre was inherently preferred by bees and most convenient for a successful landing.

In addition to this general trend, Figure 1 illustrates how flight paths were more streamlined toward bicolour stimuli than to single-colour stimuli, presumably aided by the central contrast line. It is known that contrast lines and edges are salient visual features for bees, used to steer flight behaviour (Lehrer et al., 1985, 1990). Bees approached the lower edges of the single colour discs very closely before ascending steeply toward the centre, whereas those trained to bicolour discs began to ascend slightly earlier, generally before 2 cm horizontal distance from the target, and cluster more around the target centre (Figure 1). This difference was reflected in the approach angles of the bees (Figure 1), and the statistical analysis demonstrates a significantly greater spread of steeper negative angles in response to the single-colour as compared with bicolour targets that shared the same colour in the lower half of the disc (Blue vs. Y:B: Mardia-Watson-Wheeler test, adjusted α = 0.025, W = 18.06, *p* < 0.0001; Yellow vs. B:Y: *W* = 8.08, *p* = 0.018). There was a significant difference between bicolour pattern treatments (B:Y vs. Y:B, adjusted α = 0.025: *W* = 8.7, *p* < 0.001) which could potentially result from differences in contrast strength of the lower edge.

The approach flights differed to some extent between the single-coloured blue and yellow discs (Blue vs. Yellow, adjusted α = 0.025: *W* = 16.28, *p* < 0.001): bees exhibited a similar peak in the approach angles at 20–30° below the centre line but differed in the distribution of the negative angles, which might suggest that they were also guided by the higher-contrasting lower edge in the yellow disc.

#### Colour Learning Tests

After ten training trials, we conducted unrewarded test trials to find out what the bees had learned about the colour patterns. We recorded their search behaviour on unrewarded bicoloured discs, presenting several rotations to account for a spatial bias that could arise if during training bees approach one half of the training stimulus more. Since there was no reward present during tests, the bees did not attempt to land but searched in front of a test stimulus.

**FIGURE 2:**
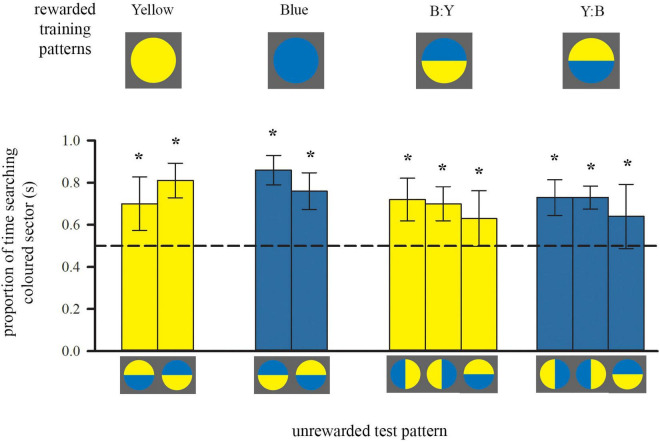
Colour preferences of trained bees in each of either two or three unrewarded tests after training to single-colour discs or bicolour patterns, respectively. Bees inspected the unrewarding test pattern whilst flying in front of it, within a distance of 5 cm. Bars represent the mean proportion of time spent flying in front of the test pattern segment that was of either the colour of the trained single-coloured disc or the colour of the lower half of the bicolour training pattern (also depicted by bar colour), relative to the search on the other segment of the test pattern. For sample sizes see Figure 1. During each test only one of the test patterns was presented. Error bars represent ± 1 standard deviation. Asterisks * above bars denote a significant deviation from equal choice at α = 0.05 (Paired *t*-tests; see also Supplementary Table 1).

Bees trained with single colours showed a significant preference for their training colour vs. a novel colour (Figure 2, left; see also Supplementary Table 1). But they did not ignore the other colour completely. This suggests that spatial cues provided by the outer edge of the disc are likely to be learned as well during the approach. Although this might influence the test performance to some degree, these cues do not dominate the behaviour of the bees which preferred the learned colour in each of the two spatial configurations in the test patterns.

After training with a bicolour pattern, bees did not search equally on both colours during tests (Figure 2, right), even though their training patterns presented equal amounts of both yellow and blue. Instead, both groups of bees showed a significant preference for the colour of the lower half of their respective training pattern: bees trained with B:Y spent significantly more time searching the yellow sectors of the test patterns for the reward, regardless of spatial position, and *vice versa* bees trained with the Y:B pattern searched more at the blue sectors (Figure 2). This preference for the yellow sector was not quite as strong as for bees trained with single-colour stimuli [ANOVA, *F*_(1_, _39)_ = 8.69, *p* = 0.005], suggesting that bees might also have perceived and learned the top colour of the training pattern during approach flights.

Similar to the groups above, trained with a single colour, it is likely that bees acquired and used spatial information, arising from the outer edge, in addition to the contrast line. However, they did not simply prefer the lower part of the test pattern with the horizontal line where the presentation of colours was inversed. This shows that they relied predominantly on the learned colour during the test. Importantly, there was also a positive correlation between the amount of time individuals spent flying below the centre of the bicolour patterns over ten training flights, and the strength of their preference for the colour of the lower half (Spearman Rank, *r*_*s*_ = 0.407, *n* = 23, *p* = 0.027).

### Experiment 2: Manipulation of the Position of the Contrast Line in Bicolour Patterns

**FIGURE 3:**
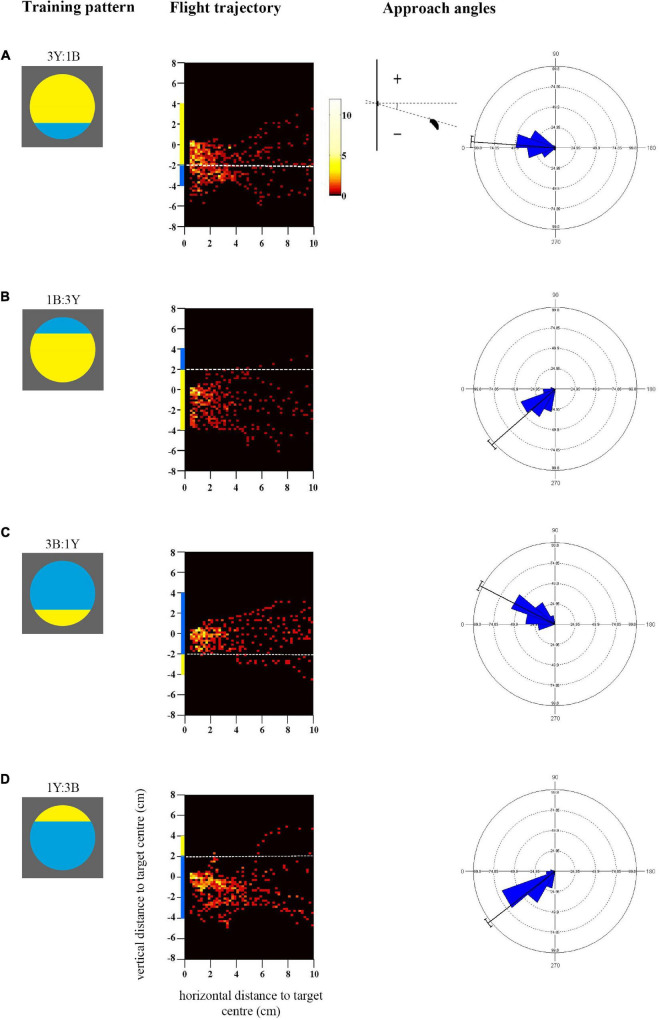
Approach flights of bees trained to collect sucrose from the centre of bicoloured patterns with an off-centre contrast line over a distance of 10 cm up to 0.5 cm. Training patterns were **(A)** 3Y:1B (mostly yellow) pattern with the contrast line and a blue segment in the bottom half or **(B)** 1B:3Y in the top half of the target, or **(C)** 3B:1Y (mostly blue) pattern with the contrast line and yellow segment in the bottom half or **(D)** 1Y:3B in the top half of the target. As in Figure 1, the heat maps depict the position of the bees during the last training flight before tests (*N* = 10 bees for all groups). White dotted lines indicate the position of the contrast line. *X*-axis depicts horizontal distance and *Y*-axis vertical distance to the target plane. Approach angle plots show the angle between the head position of the bee and the contrast line.

**FIGURE 4:**
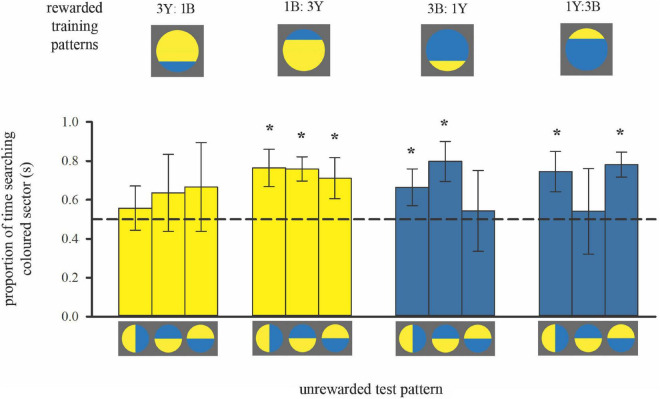
Colour preferences of bees trained with bicolour patterns with unequal segments (3:1 diameter ratio) in subsequent unrewarded tests. As in Figure 2, the search time is shown when the bees flew in front of the unrewarded pattern displaying search behaviour, within the distance of 5 cm from the target. Bars represent the mean proportion of time spent searching on the test pattern segment that displayed the same colour as the larger segment of the training stimulus (also depicted by bar colour) relative to the search on the other segment, in each of the three tests. For sample sizes see Figure 3. Error bars represent ± 1 standard deviation. Asterisks * above bars denote a significant deviation from equal choice at α = 0.05 (Paired *t*-tests; see Supplementary Table 1).

To investigate the causal relationship between approach flight and colour learning, we trained bees to one of four bicolour patterns with an unequal ratio of the two colours, where the contrast line was shifted into either the upper or lower half of the disc (Figures 3, 4). Thus, the patterns resembled the single-coloured discs in Experiment 1 with regards to the colour covering the area surrounding the reward location. The difference was that the new training patterns displayed a small segment of the second colour in either the top or bottom of the disc. If during training bees would again approach the target pattern from below centre, irrespective of the overall colour distribution, and if they ignored the segment when presented in the top of the disc and in the periphery of their visual field, we predicted that their performance in the test should be the same as after training with the single-coloured disc. We also expected that they would prefer both colours in the test when the small segment was present in the lower half of the training pattern and they approached the target from below centre during training.

#### Approach Flights During Training

The heat-maps in Figures 3A,B show that bees approaching the mostly yellow patterns spent considerable time viewing the lower half of the discs, from the centre to beneath the lower edge (see also Supplementary Figures 2C,D). When the blue segment was in the top half of the yellow pattern (1B:3Y) bees did not fly higher to view it frontally. Accordingly, they spent more time viewing the larger yellow area frontally (Mean/SE 23.1 ± 1.5 s) than the blue segment (Mean/SE 0.7 ± 0.2 s, Paired *t*-test *t* = 15.4, *p* < 0.001) during training which explains the significant yellow preference shown in the subsequent tests (Figure 4). When the blue segment was present in the lower half of the pattern (3Y:1B) it was viewed equally with the yellow area (Mean/SE yellow area 20.1 ± 1.9 s, blue area 13.3 ± 2.6 s, *t* = −1.57, *p* = 0.15), enabling the bees to see both colours with the frontal part of their eyes and associate them with the reward. There was a significant correlation between the time spent viewing the lower half of the pattern during training and the time spent on the yellow colour in the test pattern (90° rotated pattern, *r*_*s*_ = 0.52, *p* = 0.021, *N* = 19), as well as the blue colour (*r*_*s*_ = 0.64, *p* = 0.003), which is in line with the observed approach paths.

In contrast, bees trained to both mostly blue patterns flew significantly higher, closer to the height of the target centre which contained the reward [*F*_(1_, _36)_ = 8.6, *p* = 0.006; Supplementary Figure 2D]. This was particularly evident with the 3B:1Y pattern [Figure 3C, comparison with the 3Y:1B pattern (Figure 3A), Mardia-Watson-Wheeler test, *W* = 27.85, *p* < 0.001], where bees never flew below the contrast line within the last 4cm distance of the target. When the contrast line was in the upper half of the pattern (1Y:3B), bees largely approached from below centre and did not fly directly in front of the yellow segment (Figure 3D), although they appeared to be more oriented toward the centre of the disc than those trained with the reversed colour arrangement in the pattern 1B:3Y (Figure 3B, Mardia-Watson-Wheeler test, *W* = 10.19, *p* < 0.01). Both groups spent more time viewing the blue area than the yellow segment during training (Mean/SE 1Y:3B blue 22.1 ± 2.8 s, yellow 0.6 ± 0.1 s, *t* = 8.29, *p* < 0.001; 3B:1Y blue 21.2 ± 1.2 s, yellow 1.6 ± 0.4 s, *t* = 13.46, *p* < 0.001). This explains the preference for the blue colour in some of the later tests. Finally, in contrast to the bees trained with mostly yellow patterns, no correlation was observed here between the time spent viewing the lower half of the training pattern and the time spent searching on the yellow colour of the test pattern (90° rotated pattern, *r*_*s*_ = −0.42, *p* = 0.06, *N* = 20) or the blue colour (*r*_*s*_ = −0.37, *p* = 0.11). The reason for this difference could well be the more pronounced variation of approach angles and correspondingly flight height (Figures 3C,D).

#### Colour Learning Tests

Training to mostly yellow colour patterns resulted in the predicted colour preferences (Figure 4 left; see also Supplementary Table 1). When the contrast line was in the lower half of the training pattern with the smaller bottom blue (3Y:1B), bees showed no significant preference for yellow in subsequent tests with bicoloured/rotated stimuli, even though yellow was the majority colour in the training pattern. Conversely, when the contrast line was in the upper half of the training pattern (1B:3Y), bees showed a significant preference for yellow in subsequent tests.

Bees trained to mostly blue colour patterns deviated from the predicted pattern of colour preferences (Figure 4, right, Supplementary Table 1). In two out of three tests individuals had a significant preference for blue regardless of contrast line position during training, suggesting that they had learnt to associate blue more strongly with the reward. But the test pattern where colour positions were reversed, creating an “inconsistent” spatial arrangement, elicited equal colour preferences. This result implies that for the mostly blue patterns, bees predominantly viewed the blue area in front of them, but they also learnt the spatial distribution of the two colours around the contrast line. Indeed, variation in approach flight behaviour can explain this difference in learning performance when training bees to either mostly blue or mostly yellow patterns (Figure 3).

#### Relating Approach Flights and Colour Choices

Flight patterns can explain the preference for blue in two out of the three tests for bees trained with mostly blue patterns. However, when tested with “inconsistent” spatial arrangement of colours around the contrast line, the same bees showed equal preferences for both colours (Figure 4). It appears that the bees’ search behaviour in these tests was influenced not only by colour, but also by the spatial cues in the trained pattern.

The approach angles differed significantly. For the mostly blue pattern with the contrast line in the lower half (3B:1Y) there was less variation in approach angles than in the corresponding colour reversal, the pattern 3Y:1B (Figures 3A,C), as shown above. Bees seem to have utilised the contrast line to guide their approach toward the central reward by positioning themselves above it, and therefore learned the spatial distribution of the colours. However, they did not fly directly in front of the small yellow segment, and therefore might not have associated it as strongly with the reward as the blue colour. Furthermore, when the contrast line was in the top half of the pattern (Figures 3B,D) bees trained with the mostly blue pattern (1Y:3B) did not fly as low and approach as steeply as those trained with the colour-reversal (1B:3Y), suggesting that they may also have attended the contrast line to guide the approach from below.

In addition to the contrast line, the outer lines of the target could also have influenced the bees’ approach and landing manoeuvres. When measured with a spectrophotometer and modelled for the bee eye (see Methods), yellow was found to have a higher L-receptor (brightness) contrast for bees than blue against the grey background. The mostly yellow patterns provided salient edge cues that differed from the small but conspicuous yellow segment in the mostly blue patterns that were positioned either above or below the reward location. Interestingly, in the previous experiment we observed a difference in spread of approach angles between the blue and yellow single-coloured discs (Figures 1A,B) that could well be also a consequence of the outer coloured edges against the grey background.

We did not find a direct association between the frontal viewing of the main colour in the training phase of this experiment. There was no correlation between the time spent viewing the blue colour during training and the time spent on it in the test pattern (90° rotated pattern, *r*_*s*_ = −0.11, *p* = 0.65) in the mostly blue patterns and for yellow in the mostly yellow patterns (90° rotated pattern, *r*_*s*_ = 0.25, *p* = 0.303). This further aligns well with the conclusion that viewing conditions during training influenced how bees learned during their approach flight, prior to landing on the pattern.

## Discussion

In the present study we addressed the mechanistic interaction between movement and visual perception when bees approach and learn a flower-like colour pattern. We observed variations in learning performance between different colour treatments. The most parsimonious explanation is that these differences are a consequence of variations in flight behaviour prior to landing. Bees modified their approach height in response to the distribution of salient pattern cues (contrast line and salient outer edges), within narrow limits dictated by their preferred landing position, and learned the colours they were incidentally exposed to as a result of this trajectory. This suggests that flight manoeuvres during approach and landing on a vertical target influences what they learn whilst performing a foraging task at a fully visible pattern.

It has been extensively studied how walking and flying insects perform visually guided flight control and navigation tasks. Visual information is used to avoid collisions, negotiate narrow gaps, land on a surface, or locate invisible nest or foraging sites (reviewed by Srinivasan and Zhang, 2004; Egelhaaf et al., 2012). In navigation, routes will be determined by the availability of suitable visual cues (Harris et al., 2005; Collett et al., 2006; Collett, 2010; Graham and Philippides, 2017). Central-place foragers (bees and wasps) can facilitate the acquisition of visual landmark and optic flow cues required for large-scale navigation by adopting specific motor patterns (reviewed by Land and Collett, 1997; Collett and Zeil, 1998; Wystrach and Graham, 2012; Webb and Wystrach, 2016), and flexibly adjust their flight behaviour for solving spatial orientation tasks (Lehrer and Srinivasan, 1994; Lehrer, 1996). Thus, for active vision, for the necessary acquisition or use of specific visual cues, bees in some instances can modify the motor output, whereas the mechanism we describe here demonstrates how a necessary or efficient motor action can incidentally determine visual input. This deterministic effect of movement, particularly when bees learn about features of a rewarding target, provides a simple mechanism for explaining performance.

An intriguing example is the well-documented bias in bees to perform visual tasks better if stimuli are presented in the lower vs. the upper part of the frontal visual field. This “dorso-ventral asymmetry” (Wehner, 1972; Menzel and Lieke, 1983; Giurfa et al., 1999a; Lehrer, 1999) has been attributed to adaptations in central neural mechanisms for flower detection and recognition in the lower half of the bee eye, as there are no peripheral visual specialisations that could explain it. This hypothesis assumes implicitly that the dorsal and ventral visual fields of the bee are always aligned with the upper and lower halves of a target offering reward in its centre. However, the viewing conditions of individual bees will be wholly dependent upon their flight behaviour during approach and landing on the stimulus, and previous evidence suggested that bees might generally approach vertical stimuli from below (Anderson, 1977; Giger, 1996). Although the bee eye has a large field of view, which is useful for guiding movement in three dimensions, the frontal part of their compound eye has the highest visual acuity, which is best-suited for important visual tasks (Meyer-Rochow, 1981; Seidl and Kaiser, 1981; Giger and Srinivasan, 1997; Land, 1997; Graham and Collett, 2002; Hempel de Ibarra et al., 2009; Robert et al., 2017; Taylor et al., 2019). Thus, the segment of a stimulus projecting onto the frontal areas of the bee eye would primarily determine what the bee learns from their views of visual scenes, patterns or objects, and this would differ if the bee approached from different angles. Our findings indicate that this simpler, alternative explanation for dorso-ventral asymmetries in pattern learning should not be easily discarded.

The remarkable consistency of below-centre approach paths suggests that this flight trajectory is economical and convenient for landing. The bee has to land on its legs, which are positioned ventrally, such that the head is aligned with the target centre where the bee consumes the reward. Just prior to that, the flying bee must pitch the body without losing balance, from the more horizontal angle sustained during forward flight to nearly vertical (Evangelista et al., 2010; Reber et al., 2016), while continuously reducing speed (Srinivasan et al., 2000; Baird et al., 2013; Ibbotson et al., 2017). An ascending bee coming from below centre can start doing this from some distance (in our experiments from about 2 cm), and can easily accelerate and ascend if the landing has to be aborted. A descending bee coming from above centre would have to keep its body axis as horizontal as possible to reduce height and speed (Baird et al., 2013), leaving little time and space to swing the abdomen into the vertical pitch, thus risking loss of balance and failure to land. Flying straight toward the centre, pitching the body vertically whilst maintaining a straight trajectory from further away, could be more difficult, as slow flight speed and vertical posture could increase the aerodynamic drag downwards (Luu et al., 2011; Taylor et al., 2013). Bees that showed more central approaches in our experiments, for example in response to the mostly blue patterns with a low contrast line, and even the few above-centre trajectories (see Figures 1A, 3C), generally dipped down just prior to landing, in order to position the legs and head correctly. It therefore seems that the optimal way to achieve the landing position is to approach from below guided by salient pattern cues, as we find here. It is noteworthy that even when the highly salient small yellow segment was in the upper half of the colour pattern (1Y:3B pattern, see Figure 3D), the bees still did not approach above centre and specifically look at these cues. Instead, they flew slightly higher than those bees trained with the colour-reversal, sufficient to attend to the contrast line and guide the approach toward the reward location. Thus, although the bees adjusted their flight behaviour, this occurred within narrow limits, supporting the hypothesis that landing from below is easier than from above, and bees are constrained by the mechanics of this manoeuvre.

We conclude that viewing conditions are critical in determining what bees learn about visual stimuli, providing simpler explanations that should be ruled out when proposing cognitive mechanisms, such as position-weighting factors (Wehner, 1972; Thivierge et al., 2002), localised feature-extraction and expansion of the visual field (Giurfa et al., 1999a), or attentional focus (Morawetz and Spaethe, 2012). Detailed analyses of spatial behaviour may reveal that this mechanism underlies or influences performance in more complex tasks, such as recognising human faces, discriminating forest scenes, or using aesthetic sense to choose between Monet and Picasso (Dyer et al., 2005; Dyer et al., 2008; Morawetz and Spaethe, 2012; Wu et al., 2013). Morawetz and Spaethe (2012) tracked the bees that chose artificial flowers of the rewarded colour in a vertically arranged multifloral array and suggested that flight behaviour might have played a role to shape the bees’ responses, thus acknowledging that they could not fully rule out simpler explanations. A subsequent analysis of the data confirmed that bees varied the height of their flight in some of the tasks (Morawetz et al., 2014), which in line with our conclusions, and suggests that in those experiments bees reverted to simpler solutions for solving the learning task at hand. Our conclusions are further supported by a recent study in which honeybees used scanning time as an alternative, non-numerical learning strategy to solve a numerosity task. In this experiment, bees were rewarded on the stimulus that showed more black solid shapes as compared to a second, simultaneously displayed stimulus that contained fewer shapes and was laced with aversive quinine (MaBouDi et al., 2020).

There is evidence to suggest that the efficiency of the bees’ flight trajectories may be relevant for viewing and learning in more natural settings. For example, field observations commonly describe the strong directionality of bumblebees foraging on vertical inflorescences, starting at the bottom and moving upwards (Pyke, 1978; Waddington and Heinrich, 1979; Haynes and Mesler, 1984; Galen and Plowright, 1985). Flower orientation varies, and vertically presented flowers on slopes tend to adaptively face down-slope, receiving more visitation as they offer convenient petal orientation for landing of bees moving preferentially upwards (Ushimaru et al., 2006). Observations on flowers also reveal that flower orientation influences the landing behaviour of pollinators (Ushimaru and Hyodo, 2005). It is beneficial for flowers to guide pollinator movement in a way that enhances pollen transfer (Ushimaru et al., 2007; Ushimaru et al., 2009), and the fine-scale nectar guides are generally thought to function once a bee lands (Manning, 1956; Daumer, 1958; Free, 1970; Dafni and Giurfa, 1999). Flowers may exploit the tight connection between vision and movement throughout the different phases of the approach flight and landing sequence, when bees make foraging decisions. This mechanism may be decisive in how flower constancy forms when bees learn about flowers and how to handle them (Hempel de Ibarra et al., 2015).

Insect vision is inherently unintuitive; the understanding of the constraints imposed on perceptual learning, as a direct result of the animal’s own morphology and action patterns, could provide fundamental, practical, and useful insights in a field that more recently emphasises “human-like” cognitive processes. This principle is equally applicable to more familiar vertebrate groups, where species-specific viewing conditions determined by movement could provide a simple but overlooked mechanism to explain performance in perceptual learning tasks where the experimenter makes the implicit assumption that the animal views and therefore processes a given stimulus or scene in its entirety. However, viewing may differ across tasks. Birds have limited eye movements but a highly flexible neck, and so investigate objects in a very different way from mammals, by substantially moving the head to look with different parts of both eyes (Stamp Dawkins, 2002; Martin, 2007; Martin and Shaw, 2010). Head movement recruitment to shift gaze in mammals such as cats and primates is task-specific and can be blocked if energetically costly (Fuller, 1992; Oommen et al., 2004). In primates body posture and movement systematically contribute to large gaze shifts (McCluskey and Cullen, 2007). Furthermore, not all mammals have highly mobile eyes: mice, rabbit, and guinea pig do not dissociate eye and head movement much (Oommen et al., 2004). Rats are widely used in studies of visual learning, despite their relatively poor visual acuity, and often display a spatial bias for learning the lower hemifield of visual stimuli, potentially as a result of their movement along the ground and subsequent bias toward viewing the lower part of a vertical stimulus (Lashley, 1938; Minini and Jeffery, 2006). Indeed, both rats and pigeons perform significantly better in visual discrimination tasks when the targets are presented horizontally or on the floor (Delius, 1992; Furtak et al., 2009). It is likely that the movement of the animal within an experimental apparatus or structured environment will have a significant effect on the outcome of learning tasks, and experiments should be designed with ethological and morphological considerations in mind.

## Data Availability Statement

The raw data supporting the conclusions of this article will be made available by the authors, without undue reservation.

## Author Contributions

KVL, MV, and NHI conceived the experiments. KVL and CW planned and conducted the experimental work. KVL, CW, and NHI analysed the data. OR generated analysis tools. KVL and NHI wrote the original draft. All authors contributed to and approved the final version of the manuscript.

## Conflict of Interest

The authors declare that the research was conducted in the absence of any commercial or financial relationships that could be construed as a potential conflict of interest.

## Publisher’s Note

All claims expressed in this article are solely those of the authors and do not necessarily represent those of their affiliated organizations, or those of the publisher, the editors and the reviewers. Any product that may be evaluated in this article, or claim that may be made by its manufacturer, is not guaranteed or endorsed by the publisher.

## Footnotes

1
http://www.jwatcher.ucla.edu/
