# Supplementary material for: Approach Direction Prior to Landing Explains Patterns of Colour Learning in Bees
Source: Front Physiol. 2021 Dec 8;12:697886. doi: 10.3389/fphys.2021.697886 (PMC8692860; doi:10.3389/fphys.2021.697886)
Supplement: Supplementary file 1 [file Image_1.pdf]

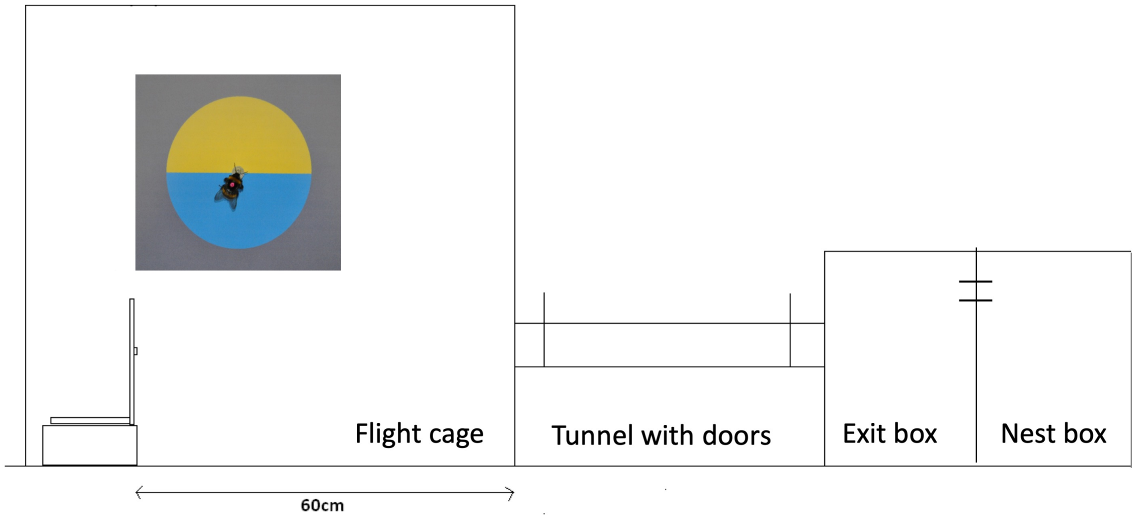

Figure S1. Schematic drawing of the setup (not to scale). Bumblebees were housed in a nest box and could access an exit box made from transparent Plexiglass. Individually-marked bees were allowed to access the flight cage (110Hx80Wx80L cm) for training and testing, through a transparent tunnel with doors that were operated manually. Only one bee was present at any time in the flight cage. Other bees from the hive were diverted from the exit box through a second tunnel into a smaller flight box containing a feeder to allow them to feed the colony (not shown). The flight cage was made from insect netting with a zipper to enable the experimenter to record from the side with a video camera that was located on a tripod outside the flight cage, or to gently catch the bee at the end of trial with a plunger tube. At the end of the trial, the bee was returned to the exit box from where she could enter the nest. Inset: During training trials the bee was rewarded at the small nib in the centre of the target stimulus on a grey background. The printed display (20x20cm) contained both stimulus and background (thus removing any potential tactile cues from the contrast and outer edges of the stimulus) and covered the vertical wall of a stand that was positioned at a distance of 60cm from the tunnel end.
