# Supplementary material for: Approach Direction Prior to Landing Explains Patterns of Colour Learning in Bees
Source: Front Physiol. 2021 Dec 8;12:697886. doi: 10.3389/fphys.2021.697886 (PMC8692860; doi:10.3389/fphys.2021.697886)
Supplement: Supplementary file 2 [file Image_2.pdf]

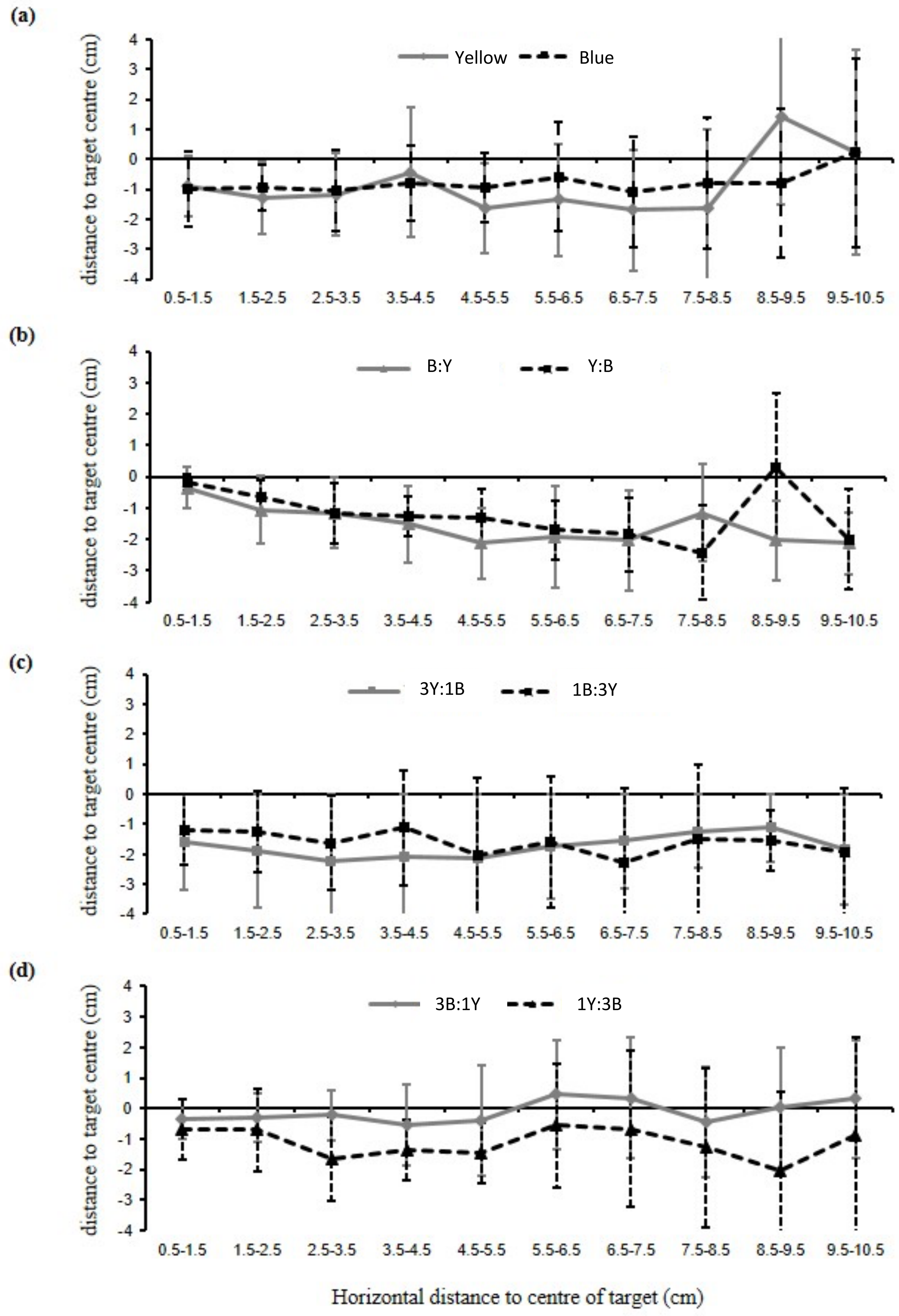
(a)
Yellow
Blue
(b)
B:Y
Y:B
(c)
3Y:1B
1B:3Y
(d)
3B:1Y
1Y:3B
distance to target centre (cm)
0.5-1.5
1.5-2.5
2.5-3.5
3.5-4.5
4.5-5.5
5.5-6.5
6.5-7.5
7.5-8.5
8.5-9.5
9.5-10.5
Horizontal distance to centre of target (cm)

Figure S2. The mean approach height of bees trained to vertical colour patterns (8cm diameter) with a sucrose reward at the centre. Data shown are for the last rewarded training flight prior to tests. (a) Single-coloured blue or yellow discs (Blue and Yellow, N=10 bees for both treatments); (b) bicolour discs with a central contrast line, with a blue top and yellow bottom (B:Y, N=11), and vice versa (Y:B, N=12); (c) with an off-centre contrast line, with the large yellow sector in the top or bottom, 3Y:1B and 1B:3Y, respectively (each N=10); (d) with an off-centre contrast line and the large blue sector in the top or bottom, 3B:1Y and 1Y:3B, respectively (each N=10). X-axis depicts horizontal distance and Y-axis vertical distance to the target plane. Error bars show standard deviation from the mean.
