# Supplementary material for: Approach Direction Prior to Landing Explains Patterns of Colour Learning in Bees
Source: Front Physiol. 2021 Dec 8;12:697886. doi: 10.3389/fphys.2021.697886 (PMC8692860; doi:10.3389/fphys.2021.697886)
Supplement: Supplementary file 3 [file Image_3.pdf]

A

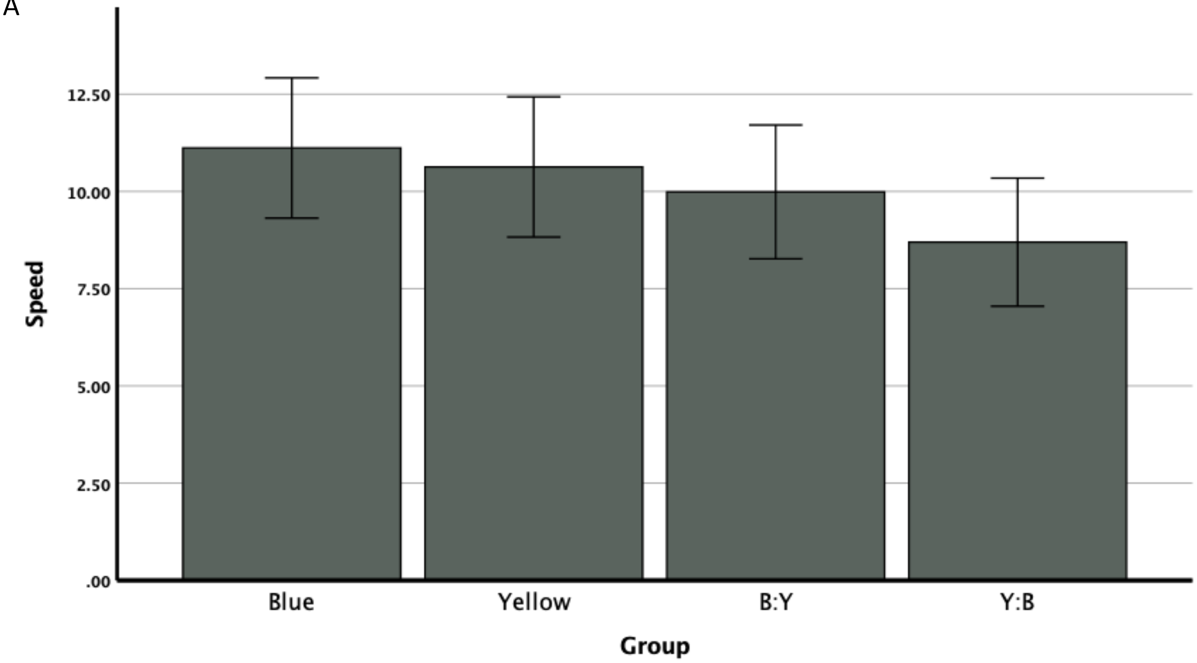

B

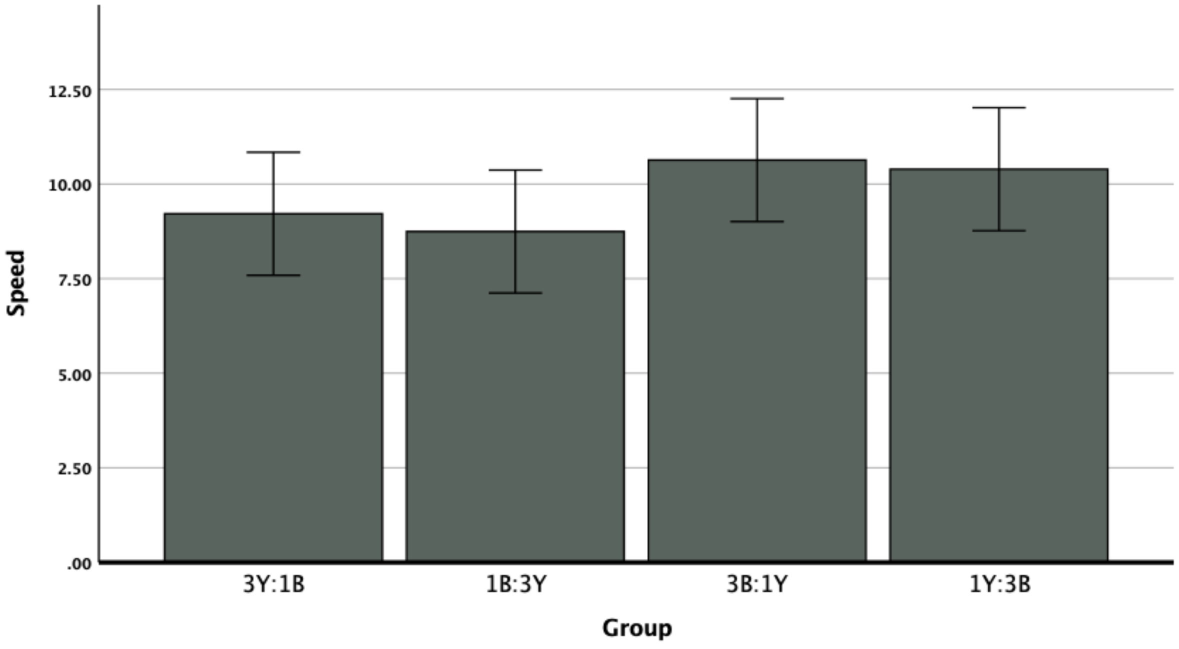

Figure S3. Average approach speeds (cm/s) in the last training trial. In Experiment 1 (A), there was no statistically significant difference in the approach speeds (ANOVA, $F(3,39)=1.507$, $p=0.228$). In Experiment 2 (B) bees also displayed similar approach speeds across groups (ANOVA, $F(3,36)=1.257$, $p=0.304$).
