# Supplementary material for: Approach Direction Prior to Landing Explains Patterns of Colour Learning in Bees
Source: Front Physiol. 2021 Dec 8;12:697886. doi: 10.3389/fphys.2021.697886 (PMC8692860; doi:10.3389/fphys.2021.697886)
Supplement: Supplementary file 4 [file Table_1.pdf]

Rewarded training pattern	N	Test pattern	Mean time (s) searching each colour ± S.E.		Paired test	Colour preference
			Blue half	Yellow half		
Yellow	10	B:Y	6.5 ±1.2	31.5 ±7.51	Z=-2.803, p=0.005	Yellow
		Y:B	8.4 ±2.17	18.2 ±3.53	t_9=-3.83, p=0.004	Yellow
Blue	10	B:Y	25.0 ±4.99	3.9 ±0.81	t_9=4.9, p=0.001	Blue
		Y:B	28.0 ±2.68	9.1 ±1.46	Z=-2.803, p=0.005	Blue
B:Y	11	90	7.6 ±1.32	18.8 ±2.01	Z=-2.803, p=0.005	Yellow
		270	6.9 ±1.56	16.3 ±2.89	t_9=-5.41, p<0.001	Yellow
		180 (Y:B)	9.5 ±2.13	17.8 ±3.9	Z=-2.09, p=0.037	Yellow
Y:B	12	90	20.1 ±3.23	7.1 ±1.18	t_{11}=4.91, p<0.001	Blue
		270	21.3 ±2.95	8.3 ±1.42	t_{11}=6.97, p<0.001	Blue
		180 (B:Y)	19.2 ±4.83	8.4 ±1.31	Z=-2.28, p=0.023	Blue
3Y:1B	10	90	17.3 ±3.53	19.6 ±3.75	t_8=-1.07, p>0.05	No
		B:Y	9.1 ±2.35	14.4 ±2.81	t_9=-1.84, p>0.05	No
		Y:B	8.6 ±7.74	18.6 ±11.74	Z=-1.78, p>0.05	No
1B:3Y	10	90	7.2 ±1.66	20.2 ±2.66	t_9=-8.71, p<0.001	Yellow
		B:Y	4.53 ±0.99	15.42 ±3.29	t_9=-4.52, p=0.001	Yellow
		Y:B	6.6 ±1.6	15.0 ±2.32	t_9=-6.5, p<0.001	Yellow
3B:1Y	10	90	17.8 ±3.92	9.5 ±2.35	t_9=3.07, p=0.013	Blue
		B:Y	21.7 ±3.47	5.6 ±1.56	Z=-2.83, p=0.005	Blue
		Y:B	14.1 ±4.44	9.3 ±1.78	t_8=1.46, p>0.05	No
1Y:3B	10	90	11.7 ±2.39	4.8 ±1.31	t_9=4.51, p=0.001	Blue
		B:Y	9.8 ±2.68	8.8 ±2.44	Z=-0.05, p>0.05	No
		Y:B	18.0 ±2.76	4.7 ±0.61	t_9=5.64, p<0.001	Blue

N = number of bees in each treatment.
Test pattern numbers refer to the degree of rotation of the training stimulus.
Paired tests give the results from t-tests when data met assumptions of normality, and Wilcoxon tests if assumptions were violated.

Table S1. Learned colour preferences of bumblebees after training to various colour patterns, differing in spatial arrangement of blue and/or yellow colour segments. Bees were trained to collect sucrose from the centre of their rewarded training pattern, and after ten trials were tested for a learned colour preference during three-minute tests with unrewarded bicoloured patterns. A colour preference is evinced by a significant difference in time spent searching one colour-half versus the other: ‘search time’ is the total time the bee spent visually exploring the test pattern within a distance of 5cm, excluding time spent landed/walking on the target.
